# Fluorescence-Tracking of Activation Gating in Human ERG Channels Reveals Rapid S4 Movement and Slow Pore Opening

**DOI:** 10.1371/journal.pone.0010876

**Published:** 2010-05-28

**Authors:** Zeineb Es-Salah-Lamoureux, Robert Fougere, Ping Yu Xiong, Gail A. Robertson, David Fedida

**Affiliations:** 1 Department of Anesthesiology, Pharmacology and Therapeutics, University of British Columbia, Vancouver, British Columbia, Canada; 2 Department of Physiology, University of Wisconsin-Madison School of Medicine, Madison, Wisconsin, United States of America; Yale School of Medicine, United States of America

## Abstract

**Background:**

hERG channels are physiologically important ion channels which mediate cardiac repolarization as a result of their unusual gating properties. These are very slow activation compared with other mammalian voltage-gated potassium channels, and extremely rapid inactivation. The mechanism of slow activation is not well understood and is investigated here using fluorescence as a direct measure of S4 movement and pore opening.

**Methods and Findings:**

Tetramethylrhodamine-5-maleimide (TMRM) fluorescence at E519 has been used to track S4 voltage sensor movement, and channel opening and closing in hERG channels. Endogenous cysteines (C445 and C449) in the S1–S2 linker bound TMRM, which caused a 10 mV hyperpolarization of the *V_½_* of activation to −27.5±2.0 mV, and showed voltage-dependent fluorescence signals. Substitution of S1–S2 linker cysteines with valines allowed unobstructed recording of S3–S4 linker E519C and L520C emission signals. Depolarization of E519C channels caused rapid initial fluorescence quenching, fit with a double Boltzmann relationship, *F-V_ON_*, with *V_½_*_,1_ = −37.8±1.7 mV, and *V_½_*_,2_ = 43.5±7.9 mV. The first phase, *V_½_*_,1_, was ∼20 mV negative to the conductance-voltage relationship measured from ionic tail currents (*G-V_½_* = −18.3±1.2 mV), and relatively unchanged in a non-inactivating E519C:S620T mutant (*V_½_* = −34.4±1.5 mV), suggesting the fast initial fluorescence quenching tracked S4 voltage sensor movement. The second phase of rapid quenching was absent in the S620T mutant. The E519C fluorescence upon repolarization (*V_½_* = −20.6±1.2, *k* = 11.4 mV) and L520C quenching during depolarization (*V_½_* = −26.8±1.0, *k* = 13.3 mV) matched the respective voltage dependencies of hERG ionic tails, and deactivation time constants from −40 to −110 mV, suggesting they detected pore-S4 rearrangements related to ionic current flow during pore opening and closing.

**Conclusion:**

The data indicate: 1) that rapid environmental changes occur at the outer end of S4 in hERG channels that underlie channel activation gating, and 2) that secondary slower changes reflect channel pore opening during sustained depolarizations, and channel closing upon repolarization. 3) No direct evidence was obtained of conformational changes related to inactivation from fluorophores attached at the outer end of S4.

## Introduction

The human *ether-a-go-go* related gene (hERG) K^+^ channel is an inwardly rectifying member of the voltage-gated potassium (Kv) channel family [1] expressed in the heart, where it forms α-subunits of the rapid delayed rectifier potassium channel, *I_Kr_* [2], [3], and makes a significant contribution to cardiac action potential repolarization. The activation of hERG channels is physiologically important in that it is relatively slow compared with other Kv channels, requiring hundreds of milliseconds to reach completion. Similarly, slow deactivation upon repolarization provides the current for termination of the cardiac action potential as the channels linger in the open state after recovering from inactivation.

The mechanism of delayed activation of hERG channels is incompletely understood but is thought to result at least partly from the slow displacement of voltage-sensing S4 segments [4], unlike other voltage-gated potassium channels which activate rapidly [5], [6], coupled to residues in the S5 and S6 domains via the S4–S5 linker [7], [8], and stabilized by charge-charge interactions within transmembrane domains [9]. Events occurring in the voltage sensor of hERG have been directly inferred from gating current recordings in the cut-open oocyte clamp, along with alanine scans of S4 [4], [10], and from the use in a single study [11] of voltage clamp fluorimetry (VCF), a technique which combines fluorescence spectroscopy to detect protein conformational changes during gating with traditional two electrode voltage clamp [5], [12], [13]. The overall suggestion of these studies is that S4 voltage sensor movement is delayed, and that this slow movement underlies the slow activation of hERG channels at physiological potentials.

Gating current studies suggest that a slow component of hERG gating charge, *Q_on_*, underlies S4 voltage sensor movement with a time constant of ∼50 ms at +10 mV which is slightly faster than channel activation, and a *V_½_* that is about 20 mV hyperpolarized compared to the conductance-voltage (*G-V*) relationship in both WT and S631A inactivation-removed channels [4]. This is an order of magnitude slower than charge movement recorded in other Kv channels [14], [15], and in eag channels [5], which makes the activation gating of hERG channels potentially unique. Fluorophores, on the other hand, attached in the S3–S4 linker and at the outer end of S4 (E518, E519, and L520) show complex emission profiles with fast and slow components that were not definitively correlated with expected charge movement [11]. The emission from L520C was unlike the gating current recordings in that the fluorescence emission showed a *V_½_* that was very close to the half-activation voltage of the *G-V*. The fluorescence signals from E518 and E519 did not correlate with channel activation, and instead were correlated with the steady state inactivation relationship.

Here, we have examined fluorescence from hERG channels during activation by labelling channels with tetramethylrhodamine-5-maleimide (TMRM) at the cysteine-substituted E519 residue. We systematically examined the E519C fluorescence report in the presence and absence of native cysteine residues in the S1–S2 linker (C445V and C449V) as no prior studies have examined the ability of these endogenous cysteines to fluoresce during activation. Our experiments revealed that after removal of the endogenous cysteines from the S1–S2 linker, separate components of the E519C:C445V:C449V fluorescence signal give excellent reports of hERG voltage sensor operation and pore coupling to ionic current activation. These results were extended using fluorescence recording from the L520C:C445V:C449V and the inactivation-removed S620T:E519C:C445V:C449V mutants, to demonstrate that hERG channel activation is accompanied by a rapid displacement of S4 detectable at E519C as a rapid voltage-dependent fluorescence quenching that precedes pore opening.

## Materials and Methods

### Ethics Statement

All animal protocols were performed in accordance with University of British Columbia animal care guidelines which conform to regulations set out by the Canadian Council of Animal Care (CCAC).

### Molecular Biology and RNA Preparation

**Figure 1:**
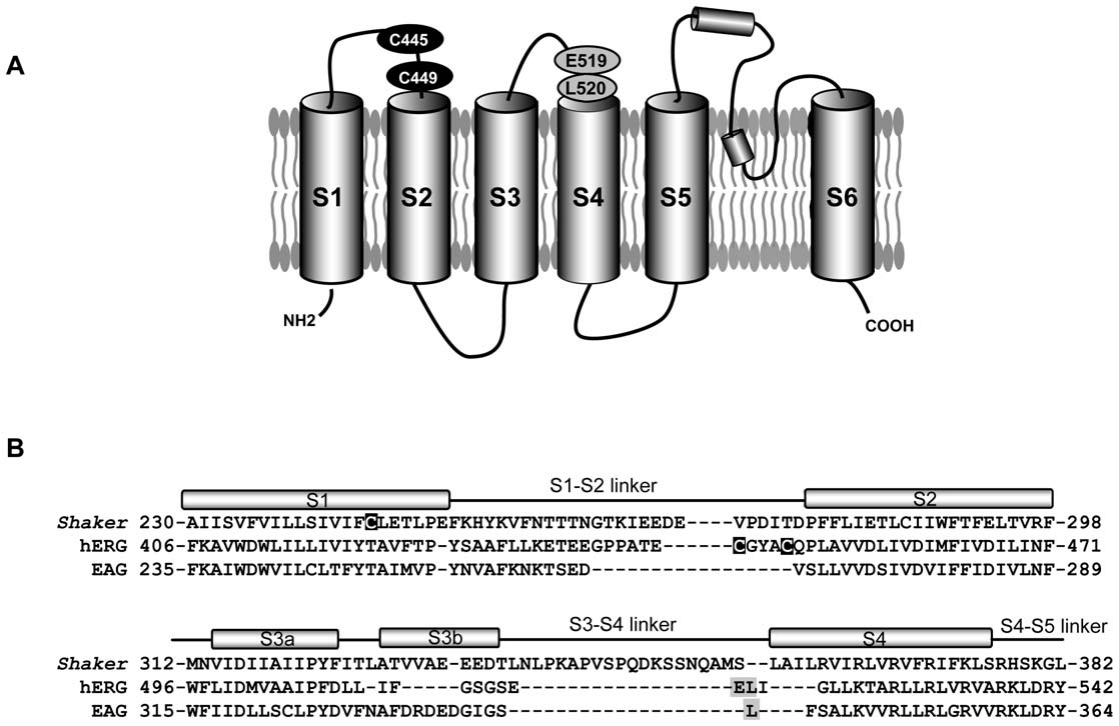
Topology of the hERG channel and location of endogenous cysteine residues. (A) Proposed membrane topology of the hERG channel showing relative positions of endogenous cysteine residues in S1–S2 linker (C445 and C449), and location of introduced cysteines (E519, L520C). (B) Alignment of primary sequences of amino acids of hERG, Shaker and EAG, in the S1–S2 and the S3–S4 regions. The exact positions of the helices and linkers are from [44]. The endogenous cysteine residue removed in Shaker fluorimetry studies (C245) and the accessible cysteine residues in the hERG channel are in white font on dark background. Black letters in gray background indicate sites of introduced cysteine residues and TMRM binding in the hERG (this study) and EAG channels [5].

hERG constructs were expressed in *Xenopus* oocytes using the pBluescript SK expression vector. Cysteines were introduced by site-directed mutagenesis in the S3–S4 linker at E519 and L520. It is difficult to correlate residues in the short S3–S4 linker of hERG with equivalent residues in the long linker of *Shaker* (Fig. 1B), but the glutamate residue is in a similar location to M356 and A359 in *Shaker*-IR channels that have been used to study activation gating in that channel [12], [13], and which produce a reliable report of voltage sensor movement during activation in Kv channels [16], [17]. The L520 residue is aligned with L342 in eag channels that also gives a large voltage-dependent fluorescence signal [5]. Two endogenous extracellular cysteines (C445 and C449) are present in hERG channels in the S1–S2 linker (Fig. 1). This is somewhat different from *Shaker* channels where a cysteine is located at the extracellular end of S1, as shown in the alignment of the two channels in Fig. 1B.

For mutagenesis, oligonucleotide primers were synthesized by Integrated DNA Technologies (Coralville, IA), and mutations were generated using the QuikChange II site-directed mutagenesis kit (Stratagene, La Jolla, CA). Successful mutations were confirmed by sequencing the constructs using the core facility unit at the University of British Columbia or by Macrogen (Seoul, Korea). cDNA was linearized with *Not*I for RNA transcription. cRNA was synthesized from linear cDNA using the mMessage mMachine T7 Ultra transcription kit (Ambion, Austin, TX) and collected using the RNeasy micro kit (Qiagen, Valencia, CA) at a concentration ranging from 500 to 1500 ng/µL.

### Oocyte Preparation and Injection

*Xenopus laevis* frogs were terminally anesthetized by immersion in 2 mg/mL tricaine methanesulphonate (Sigma-Aldrich, Mississauga, ON, Canada); unless otherwise stated, all chemicals were purchased from Sigma-Aldrich. Stage V-VI oocytes were isolated and enzymatically defolliculated using a solution of (in mM), 82.5 NaCl, 2.5 KCl, 1 MgCl_2_, and 5 HEPES with 2 mg/mL type 1a collagenase (Sigma) for approximately 1 hour in combination with orbital shaking. Between defolliculation and injection, oocytes were incubated for 1–18 hours in a modified oocyte Ringer's solution, which contained 500 mL Leibovitz's L-15 Medium (Gibco) and (in mM), 15 HEPES, 0.52 gentamicin (Gibco), 1 L-glutamine, titrated to pH 7.6 using NaOH. Injection of 50 nL of cRNA was performed using a Drummond microdispensor (Fisher Scientific, Ottawa, ON, Canada), followed by incubation at 19°C. VCF recordings were performed 3 to 5 days after injection.

### Two-electrode voltage clamp

Oocytes were placed in a bath chamber that was perfused with control ND96 bath solution containing (in mM), 96 NaCl, 3 KCl, 1 MgCl_2_, 2 CaCl_2_, and 5 HEPES, titrated to pH 7.4 with NaOH. Experiments at pH 5.5 were performed in ND96 solution substituting 5 mM MES for HEPES. Microelectrodes were filled with 3 M KCl and had resistances of 1 to 5 MΩ. Voltage control and data acquisition was achieved with a Warner Instruments OC-725C amplifier (Hamden, CT), and Axon Digidata 1322 A/D converter (Axon Instruments, Foster City, CA), connected to a personal computer running pClamp9 software (Molecular Devices Corp.).

### Voltage clamp Fluorimetry

Fluorimetry was performed simultaneously with two-electrode voltage clamp. Labelling of the oocytes with tetramethylrhodamine-5-maleimide (TMRM; Invitrogen, Carlsbad, CA) dye was performed at 10°C in a depolarizing solution containing (in mM), 100 KCl, 1.5 MgCl_2_, 0.5 CaCl_2_, and 10 HEPES, titrated to pH 7.4 using KOH, with 5 µM TMRM. After 30 minutes of labelling, oocytes were stored in ND96 solution in the dark until voltage clamped. Fluorimetry was performed using a Nikon TE300 inverted microscope with Epi-Fluorescence attachment and a 9124b Electron Tubes photomultiplier tube (PMT) module (Cairn Research, Kent, UK) as we have described previously [17]. To minimize fluorophore bleaching, a Uniblitz computer-controlled shutter (Vincent Associates, Ottawa, ON, Canada) was used, and opened shortly prior to application of voltage clamp pulses. Fluorescence signal sampling frequency was 6.67 kHz; when averaged, signal traces represented an average of 2–7 sweeps, and were filtered offline at 300–1000 Hz. To correct for photobleaching of fluorophore that occurred during shutter opening during activation protocol and single sweep experiments, control fluorescence data were recorded in the absence of any change in voltage, and subtracted from the voltage-dependent signal.

### Data Analysis

Conductance-voltage (*G-V*) relationships were derived by plotting peak tail currents as a function of the preceding depolarizing pulse. Fluorescence-voltage (*F-V*) curves were derived as stated in the figure legends. *G-V* and *F-V* curves were fit with a single Boltzmann function:
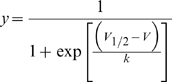
Or a sum of two Boltzmann functions:
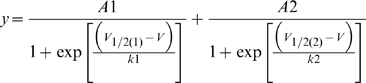
Where *y* is the Δ fluorescence or the conductance normalized with respect to the maximal Δ fluorescence or conductance, *V_1/2_* is the half-activation potential, *V* is the test voltage, and *k* is the slope factor. *A* refers to the amplitude of the fit component, and *1* and *2* refer to the separate components of the fit. Unless otherwise indicated, data reported throughout the text and figures are presented as mean ± SEM.

## Results

### Endogenous fluorescence from WT hERG channels

Previous work with *Shaker* channels looking at TMRM fluorescence from sites at the outer end of S4 has always mutated C245, located at the top of S1 segment, to valine to prevent mistargeted labelling [12], [13], [18]. Based on sequence alignments and homology modeling, four cysteines are potentially available for labelling in hERG. They are: C445 and C449 in the S1–S2 linker, C566 in the S5 domain and C643 in the S6 domain. The residues located in the S5 and S6 transmembrane domains, C556 and C643, are most likely inaccessible to TMRM, so initial experiments focused on C445 and C449 (Fig. 1).

**Figure 2:**
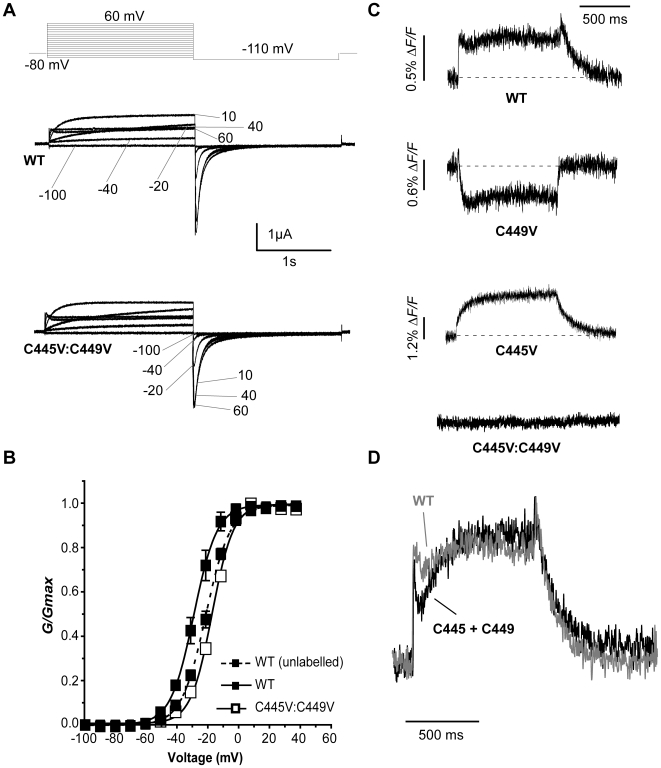
Endogenous cysteine residues are accessible to TMRM labelling. (A) Representative hERG currents (WT and C445V:C449V) evoked by 2 s depolarizing steps from −80 to potentials ranging from −100 to 60 mV. Membrane was then hyperpolarized at −110 mV for 2 s. (B) Comparison of WT and C445V:C449V *G-V* curves with (solid line) and without (dotted line) TMRM labelling, obtained by plotting the maximum tail current against voltage (WT unlabelled *V_½_* = −19.7±1.2 mV and *k* = 8.1±0.2, n = 5; WT labelled *V_½_* = −27.5±2.0 mV and *k* = 7.7±0.7, n = 3; C445V:C449V *V_½_* = −15.7±0.7 mV and *k* = 7.1±0.3, n = 6). (C) Representative TMRM fluorescence traces recorded during 1 s depolarizations from −80 to +40 mV and then repolarized to −110 mV from: WT channels, channels with one cysteine (C449V, C445V), and channels with both cysteines removed (C445V:C449V). Note that C445V:C449V channels no longer fluoresce. (D) Arithmetic addition of fluorescence reports from C445V and C449V (black) compared to the WT signal (gray).

The first indication of involvement by native cysteines was a hyperpolarizing shift in the *G-V* of WT hERG upon TMRM labelling (*V_½_* = −27.5 mV in labelled, and −19.7 mV in unlabelled channels respectively, Fig. 2B). This suggested that TMRM was able to access and bind to endogenous cysteine residues in the channel. Therefore, we looked to see if there was an effect of TMRM labelling on the voltage dependence of activation of hERG channels with the S1–S2 linker cysteines mutated to valine. Currents in WT and C445V:C449V hERG channels are shown in Fig. 2A, and appear little different. Channels expressed at similar levels to WT and showed the usual negative slope conductance of steady state currents due to rapid inactivation and large deactivating tail currents on repolarization to −110 mV. After replacement of C445 and C449 with valines, the *G-V* relationship was unchanged after labelling (*V_½_* = −15.7 mV) and close to that of unlabelled WT channels (Fig. 2B). These data supported the idea that TMRM could bind one or both of the S1–S2 linker cysteines, and that these are the major sites of endogenous labelling in the WT channel.

Fluorescence recording was attempted from these cysteines, with representative traces shown in Fig. 2C. The WT, C445V and C449V hERG channels all showed clear fluorescence reports during depolarizations to +40 mV (Fig. 2C). The double-mutant C445V:C449V, which eliminates both extracellular cysteine residues, did not show any fluorescence deflections. Taken together with the shift of the *G-V* relationship, the fluorescence results from TMRM labelling confirm that the extracellular S1–S2 cysteines are the only source of endogenous fluorescence in hERG channels and that neither C566 nor C643 are labelled using these procedures. We also tested the idea that the WT fluorescence signal could be formed by the composite fluorescence of the individual C445 and C449 emissions. As shown in Fig. 2D, representative C449V and C445V tracings can be arithmetically summed to yield a signal that is qualitatively identical to the WT fluorescence output. However, compared to the individual C445V and C449V signals, the composite WT signal was, on average, weaker. This might be due to the proximity of TMRM molecules attached to C445 and C449, resulting in a degree of self-quenching [19].

### Endogenous fluorescence from WT hERG channels modifies the signal from E519C

**Figure 3:**
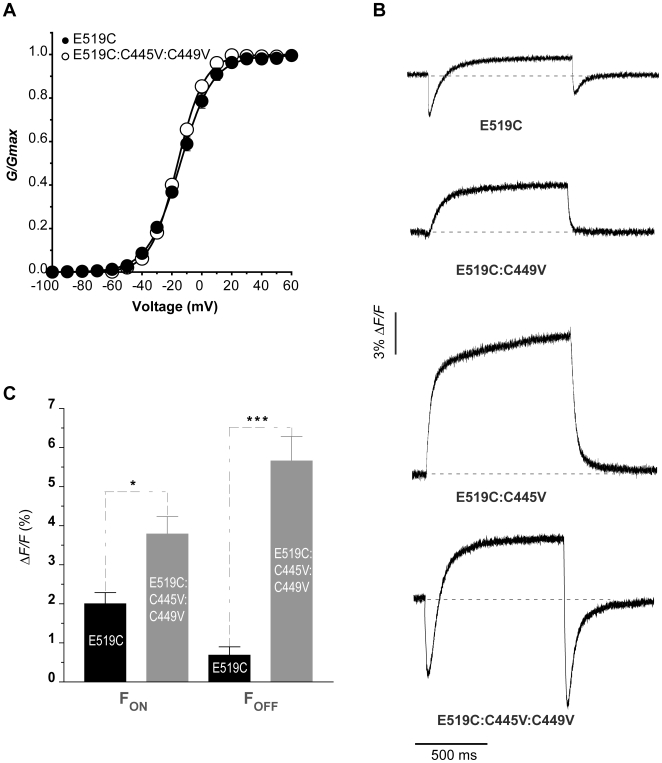
The fluorescence report from E519C is affected by the presence of endogenous cysteine residues. (A) Comparison of E519C (filled circles) and E519C:C445V:C449V (open circles) *G-V* curves. (E519C *V½* = −15.2±1.6 mV and *k* = 10.6±0.5, n = 8; E519C:C445V:C449V *V½* = −18.3±1.2 mV and *k* = 8.8±0.2, n = 7). In both cases, channels are labelled with TMRM. (B) Representative fluorescence profiles obtained with a depolarizing pulse to +40 mV for 1 s, from a holding potential of −80 mV and returning to −110 mV, comparing the fluorescence signal previously reported for E519C with signals obtained when one or both native cysteines are removed. (C) Graph showing mean fluorescence amplitudes of the initial quenching of E519C (n = 5), and E519C:C445V:C449V (n = 16), and the quenching upon repolarization (* = p<0.05; *** = p<0.0005). “*F_ON_*” and “*F_OFF_*” represent the amplitude of the fluorescence quenching upon depolarization and repolarization, respectively. Data from the two constructs were obtained from the same oocyte batches and recorded the same day.

We next examined how the presence of the cysteines at C445 and C449 positions could modify the S4 voltage sensor report from a cysteine placed at E519 in the S3–S4 (Fig. 3). It is known that replacement of E519 with alanine does not affect the Ca-independent voltage dependence of activation or inactivation [20], and we found little effect of cysteine placement at E519. Panel A illustrates that the E519C channel *G-V* relationship was almost unaffected by the removal of native cysteines. The *V_½_* of activation was −15.2 mV in E519C hERG and −18.3 mV in E519C:C445V:C449V after labelling. The fluorescence report from the E519C channel is shown at the top of Fig. 3B, and was very similar to that previously reported [11]. During a 1 s depolarization from −80 mV to +40 mV, an initial fluorescence quenching was seen at the onset of depolarization; this was followed by a fluorescence recovery above baseline and a small rapid fluorescence quenching upon repolarization to −110 mV. Representative fluorescence emission from E519C with one or the other of the S1–S2 linker cysteines replaced by valines is shown below, and it can be seen that there was a striking modification of the fluorescence output. Replacing both S1–S2 linker cysteines by valines revealed the unadulterated fluorescence emission of E519C (bottom tracing in Fig. 3B). The overall signal is now about two-fold larger on average (Fig. 3C), with a much larger fluorescence quenching upon final repolarization to −110 mV relative to the initial fast quenching observed upon depolarization. The mean data from similarly injected oocytes in Fig. 3C highlights the differences in the amplitudes of the peaks. It can be seen that the presence of the native cysteines remaining in the E519C channel attenuated significantly all aspects of the signal, most prominently the rapid quenching upon repolarization to −110 mV.

### Do cysteine-removed channels recapitulate slow activation

**Figure 4:**
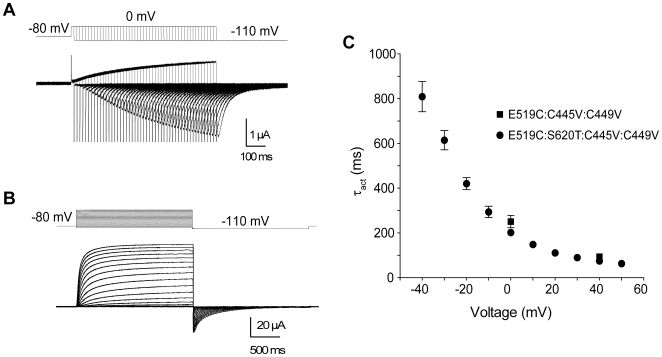
Time course of activation is slow in hERG channels. (A) Representative current traces recorded using an envelope of tails protocol, obtained from the E519C:C445V:C449V mutant. Membrane potential was stepped to 0 mV from −80 mV for varying durations of time and repolarized to −110 mV. The downward pulses are capacitive currents for each pulse. (B) Representative current traces from the E519C:S620T:C445V:C449V channels, using the same two-pulse protocol as in Fig. 2A. (C) Plot of time constants of activation against voltage. Squares represent τ_act_ from E519C:C445V:C449V for two voltages (0 and +40 mV), obtained by plotting the peak tails *versus* test pulse duration (n = 8 for each voltage), then fitting using a single exponential. Circles represent τ_act_ from E519C:S620T:C445V:C449V, obtained by fitting the current activation phase with a single exponential (n = 8).

The principal issue addressed in this paper is the measurement of direct S4 voltage sensor translocation via fluorescence environment change at the outer end of S4, without interference from endogenous cysteines in the S1–S2 linker. It is important to establish that hERG channels still activate at the same rate as WT channels after removal of the endogenous cysteines, while including the fluorophore residue used to measure S4 translocation at E519C. It is problematic to measure activation time constants at positive potentials in the presence of rapid inactivation which truncates the activation time course, so this experiment was carried out both in intact (using an envelope of tails protocol) and inactivation-removed S620T channels (using a single-pulse protocol) (Fig. 4A,B). Activation time constants (τ_act_) from the two constructs are shown in Fig. 4C and illustrate the extremely slow time course of activation of all hERG channels at all potentials, with τ_act_>200ms at 0 mV. In both E519C:C445V:C449V and E519C:S620T:C445V:C449V channels, τ_act_ was still greater than 50 ms at positive potentials, and these data are comparable with published data from WT hERG channels [9], [21]. These experiments demonstrate the validity of the cysteine-removed E519C mutant channel used to investigate the speed of S4 translocation.

### Relating two components of the E519C fluorescence signal to channel gating

**Figure 5:**
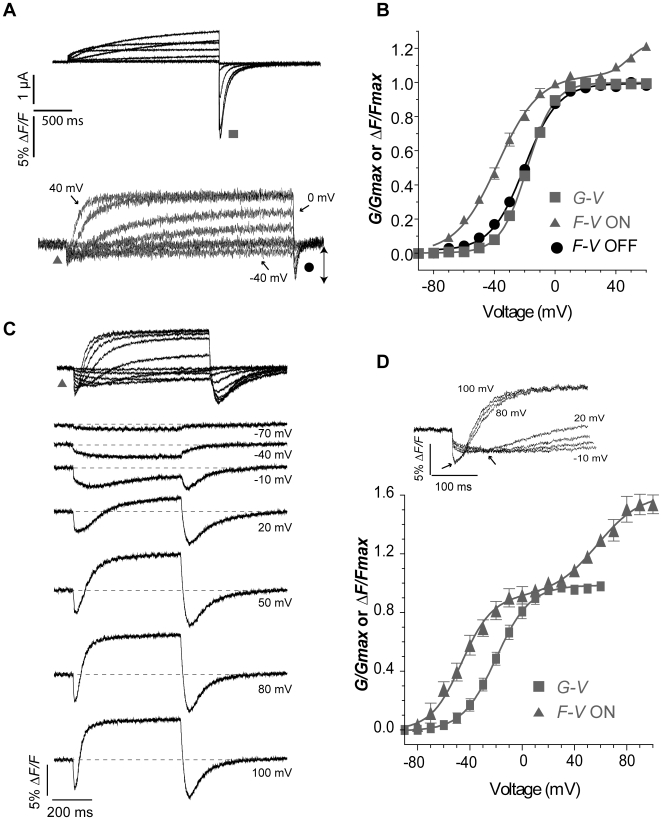
Relationship between E519C:C445V:C449V fluorescence signal and ionic conductance. (A) Representative current (above) and fluorescence traces (below) recorded using the same protocol as in Fig. 2A. (B) Comparison of *G-V* curve (squares, *V½* = −18.3±1.2, *k* = 8.8±0.2 mV, n = 7) with fluorescence-voltage relationship from the tail of fluorescence (circles, *F-V_OFF_*; *V_½_* = −20.6±1.2 mV, *k* = 11.4±0.6 mV, n = 16), measured at the times indicated by the symbols in panel A, and with fluorescence-voltage relationship from the peak fluorescence quenching upon depolarization (*F-V_ON_*, triangles in panel A). The *F-V_ON_* curve was obtained by plotting the maximum amplitude of the downward deflection for each trace, and normalized to +20 mV (estimated saturation voltage), and was best fit with a double Boltzmann function, as a second component appears at positive potentials (first phase: *V_½,1_* = −37.8±1.7 mV, *k_1_* = 13.7±0.7 mV; *F-V_ON_* second phase: *V_½,2_* = 43.5±7.9, *k_2_* = 9.6±2.6 mV, n = 13; *A*1 = 0.96±0.2). (C) Representative fluorescence traces obtained during 500 ms depolarizing steps from −80 to potentials ranging from −90 to 100 mV. Membrane was then hyperpolarized at −110 mV for 500 ms. (D) Normalized *G-V* relationship (squares, *V½* = −19.6±2.0, *k* = 13.8±2.7 mV, n = 3) obtained as in A compared to *F-V_ON_* (first phase: *V_½,1_* = −46.7±2.1 mV, *k_1_* = 12.7±1.1 mV; *F-V_ON_* second phase: *V_½,2_* = 56.7±7.2, *k_2_* = 16.9±3.9 mV, n = 3; *A*1 = 0.56±0.05). The inset shows the fluorescence signal from a representative cell, to highlight the first saturation step at about 0 mV and the second saturation step at about 80 mV.

Our hypothesis was that when C445 and C449 in the S1–S2 linker are removed, then introduced cysteines at the outer end of S4 or in the S3–S4 linker report S4 translocation during voltage-dependent activation. Replacing both S1–S2 linker cysteines by valines revealed the unadulterated voltage-dependent fluorescence emission of E519C (Fig. 5A and 5C). In initial experiments from a holding potential of −80 mV, three major voltage-dependent components of the E519C:C445V:C449V signal were observed as shown in Fig. 5A and C. These are: the initial rapid fluorescence quenching upon depolarization (▴), an exponential fluorescence increase during the course of the depolarization, and the large fluorescence quenching upon repolarization (•).

Rapid initial fluorescence quenching from S4 reporters during step depolarizations approximated the time course and voltage-dependence of gating charge movement in *Shaker* [12], [13], and also Kv channels [18], [22], suggesting that these rapid changes in fluorescence reflect conformational changes at the outer end of S4, that underlie activation gating in response to changes in transmembrane potential.

The initial quenching (*F-V_ON_*, Fig. 5B) curve was best fit by a double Boltzmann function with one component hyperpolarized compared to the *G-V* (*F-V_½_*_,1_ = −37.8±1.7 mV and *G-V_½_*_,_ = −18.3±1.2), as generally expected for a signal reporting on a charge translocation event that precedes channel opening, and another component that appears at potentials more positive than 30 mV. In order to better reveal this right-shifted portion, we used the same protocol as above, but decreased the depolarization and hyperpolarization times to 500 ms for a better resolution of the signal, and added depolarization steps to +100 mV (Fig. 5C, D). Again, a double Boltzmann was used to best fit the curve, and two saturation regions are now discernible (see inset to Fig. 5D), with the second component saturating near 90 mV (*F-V_½_*_,2_ = 56.7±7.2 mV). The underlying basis for the second, more positive component of S4 fluorescence is much less obvious, but it is interesting to note that it has a similar voltage dependence to that of the fast component of gating charge observed by Piper *et al.* [4] which has a *V_½_* of +28±4.4 mV.

**Figure 6 pone-0010876-g006:**
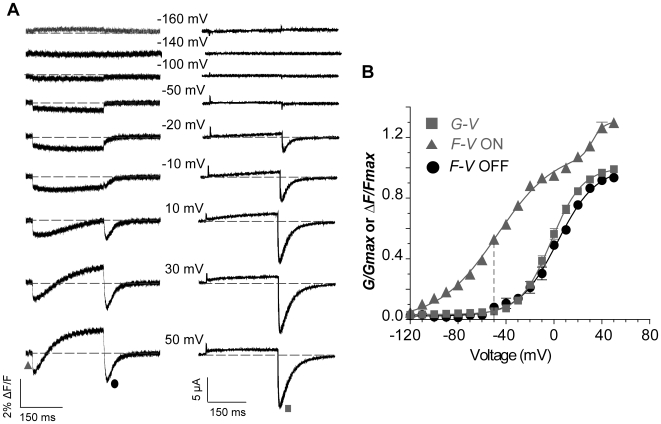
Detection of S4 movement from E519C labelling at potentials negative to channel activation. (A) Representative fluorescence and current traces recorded during 300 ms pulses from a holding potential of −140 mV to potentials ranging from −160 to 50 mV. (B) Comparison of *G-V* curve (squares, measured from ionic tail currents in A; *V_½_* = −2.2±2.2 mV, *k* = 12.8±0.2 mV, n = 3) with fluorescence-voltage relationship from the initial quenching (triangles) measured as in Fig. 5B. *F-V_ON_* curve was best fit with a double Boltzmann function, with *F-V_ON_* first phase *V_½,1_* = −48.5±2.4 mV, *k_1_* = 23.8±0.1 mV, and *F-V_ON_* second phase *V_½,2_* = 30.0±5.2 mV, *k_2_* = 4.2±1.4 mV, n = 3 (*A*1 = 0.83±0.01). Note that between −120 and −50 mV no ionic current was observed, but a fluorescence deflection could be observed positive to −110 mV. The *F-V_OFF_* is also shown (circles in panel A, *V_½_* = 2.5±1.8 mV, *k* = 16.2±2.3 mV, n = 3).

It is expected that voltage sensor movement has a more negative voltage-dependence than pore conductance, since activation of voltage sensors must occur before channel opening can occur [14], [23], [24], and this is observed in fluorescence reports from both *Shaker* [12], [13] and other Kv channels [17]. Thus, the first component of the *F-V_ON_* is a good candidate for the voltage-dependence of S4 movement. In order to examine the full extent of S4 movement at negative potentials, the experiment shown in Fig. 5 was repeated from a holding potential of −140 mV (Fig. 6). The initial fluorescence quenching, seen as a downward deflection on the emission records in Fig. 6A, first appeared during depolarizations at about −100 mV, at much more negative potentials than ionic current. Interestingly, an upward deflection is observed at −160 mV, suggesting that the fluorophore at the top of S4 segment is directed towards a more hydrophobic environment upon hyperpolarization. The first appearance of ionic conductance correlated well with the appearance of the fluorescence tail on repolarization in the records in Fig. 6A. As before, a double Boltzmann curve was fit to the initial quenching data (Fig. 6B), with a *V_½,1_* = −48.5 mV for the major component of *F-V_ON_*, well negative to the *V_½_* of the *G-V* (−2.2 mV), and with a relatively shallow voltage dependence, *k* = 23.8 mV.

### Removal of inactivation better reveals movement of S4

**Figure 7:**
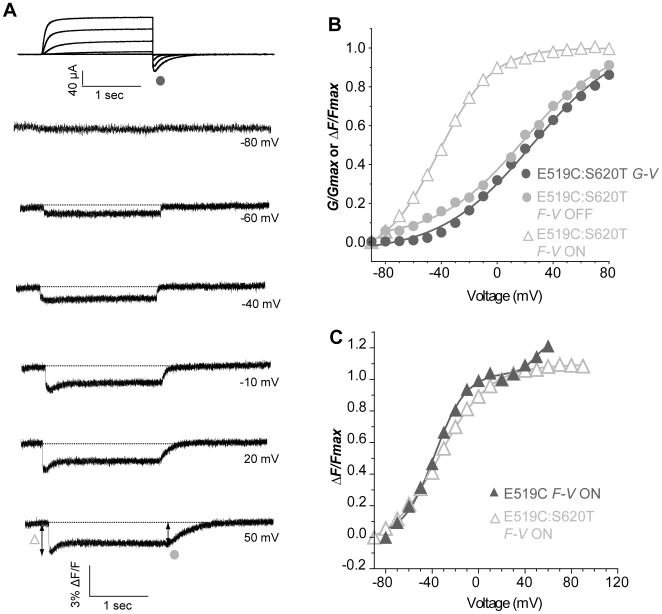
Conservation of the first component of the initial fluorescence quenching in the S620T, inactivation-removed mutant. (A) Representative traces of current and fluorescence from E519C:S620T:C445V:C449V mutant. The protocol used is the same as in Fig. 2A, but depolarization steps were applied from −100 to +120 mV (B) *G-V* and *F-V* relationships obtained for the representative cell in A. *G-V*: *V_½_* = 24.9, *k* = 32.9 mV; *F-V_ON_*: *V_½_* = −42.1, *k* = 19.3 mV; *F-V*_OFF_: *V_½_* = 18.4, *k* = 30.4 mV. (C) Comparison of mean data for the *F-V_ON_* of E519C:S620T:C445V:C449V (open triangles), *V_½_* = −34.4±1.5, *k* = 22.2±1.4 mV (n = 10) with *F-V_ON_* of E519C:C445V:C449V (closed triangles) from Fig. 5B.

In order to better understand and isolate the components of the E519C fluorescence signal, we recorded fluorescence from an inactivation-removed channel, S620T:E519C:C445V:C449V [25] as shown in Fig. 7. The fluorescence emission signal appeared less complex than before with a quenching during the step depolarization and recovery on repolarization (Fig. 7A). Importantly, upon depolarization, the initial fast downward deflection was still present, as in the intact channel, and this is further evidence to support the hypothesis that fast quenching changes in fluorescence are related to S4 movement, as opposed to inactivation processes. There is a reduced secondary increase of fluorescence during the depolarizing pulse, and this reflects a reduced ability of the fluorophore to detect S4-pore coupling during channel opening, likely as a result of changed environment around E519C in the S620T mutant. The *F-V* relationship obtained by plotting the rapid initial quenching against voltage was hyperpolarized compared to the *G-V* relation (Fig. 7B). Surprisingly, the curve was now best-fitted with a single Boltzmann function with a *V_½_* = −42.1 mV, 67 mV more negative than the *G-V* relationship for this construct (*V_½_* = 24.9 mV). The voltage dependence of the S620T:E519C:C445V:C449V construct also overlaid the first component of the double Boltzmann fit of the *F-V_ON_* relation of the E519C:C445V:C449V fluorescence signal (Fig. 7C). It appears that removal of inactivation has concomitantly removed the second Boltzmann component of fast fluorescence quenching seen at positive membrane potentials.

The *F-V* relation of the fluorescence tail on repolarization overlaid the *G-V* relation (*F-V_OFF_ V_½_* = 18.4 mV, Fig. 7B) in agreement with all data we have obtained to date that suggests that ionic current deactivation is the event tracked by the fluorescence tail on repolarization. This correspondence was further investigated as described below.

### The fluorescence quenching upon repolarization from E519C and L520C is a faithful report of pore closing and can be used to track pore opening

**Figure 8:**
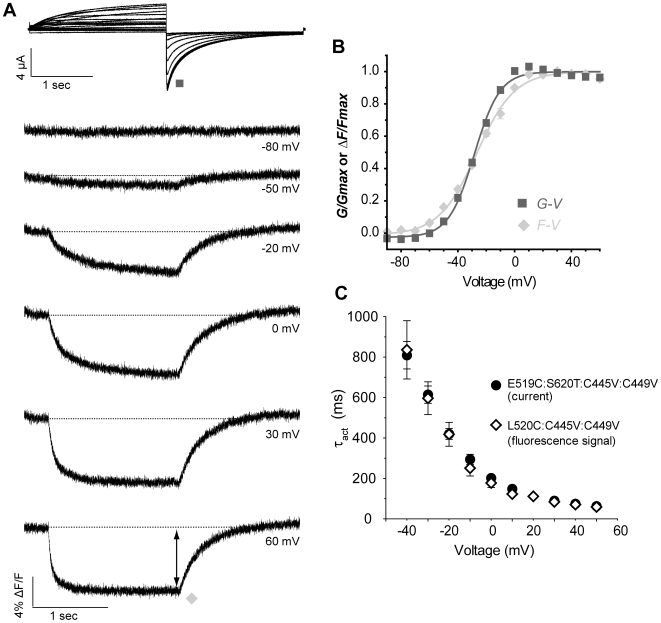
Relationship between L520C:C445V:C449V fluorescence signal and ionic conductance. (A) Representative current and fluorescence traces recorded using the same protocol as in Fig. 2A. (B) The *F-V* relationship was obtained by plotting the amplitude of fluorescence at the end of the depolarization pulse against voltage (diamonds; *V_½_* = −26.8±1.0 mV, *k* = 13.3±0.6 mV (n = 10)), and is compared to mean data for *G-V* (squares; *V_½_* = −28.4±0.7 mV, *k* = 9.0±0.2 mV (n = 6)). (C) Comparison of time course of activation of E519C:S620T:C445V:C449V channels (circles, from Fig. 4C) with the time course of fluorescence deflection from L520C:C445V:C449V mutant (diamonds, n = 6). The fluorescence signal upon depolarization was fitted with a single exponential and plotted against voltage.

Data in Figs. 5B and 6B showed the *F-V_OFF_* and *G-V* of the E519C:C445V:C449V construct at maximum quenching and peak inward current values, following repolarization (Fig. 5B: *G-V_½_* = −18.3±1.2 mV and *F-V_½_* = −20.6±1.2 mV; Fig. 6B: *G-V_½_* = −2.2±2.2 mV and *F-V_½_* = 2.5±1.8 mV). There was a strong overlap of the voltage dependence of these two components, suggesting that this quenching event may track channel pore closing. After removal of inactivation in S620T, the same correlation was observed (Fig. 7B). It was found that the fluorescence upon depolarization from a second S4 reporter, L520C, with endogenous cysteines removed, also tracked pore opening and closing closely (Fig. 8). Indeed, the *F-V* of L520C:C445V:C449V measured at the end of the depolarization had a *V_½_*, of −26.8±1.0 mV, which overlaid the *G-V* relationship for that mutant (*V_½_* = −28.4±0.7 mV) (Fig. 8B). Moreover, the time course of fluorescence signal quenching for that mutant correlates well with the time course of activation of hERG channels obtained in Fig. 4C, for the range of voltage tested (Fig. 8C)

**Figure 9:**
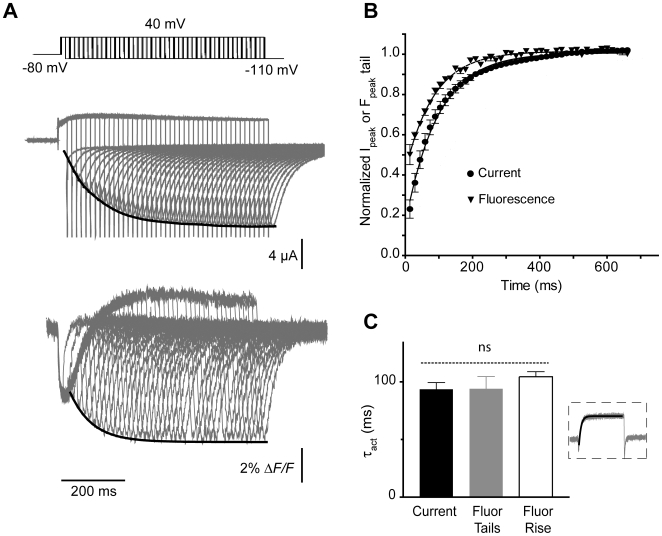
Analysis of activation time course of E519C:C445V:C449V channels and correlation with fluorescence signal. (A) Representative current and fluorescence traces, showing time course of activation measured using an envelope of tails protocol. Membrane potential was stepped from −80 to +40 mV for increasing time periods (15 to 675 ms, with 15 ms intervals), then repolarized to −110 mV. (B) The peak tail current (circles) and fluorescence (triangles) tails, obtained after repolarization from +40, were normalized and plotted against the pulse duration. (C) Time constants of activation of current (black bar) and fluorescence (gray bar), obtained from an exponential fit to the peak tails (represented by black lines in Fig. 8A). The white bar represents the exponential fit of the fluorescence deflection upon depolarization for 2 s to +40 from −80 mV (shown in inset). No significant differences were observed between groups.

An envelope of tails protocol was used to correlate E519C:C445V:C449V fluorescence to the rate of channel opening, focusing on the tail of fluorescence upon repolarization (Fig. 9). Voltage clamp pulses of increasing duration were given from a holding potential of −80 to +40 mV, followed by repolarization to −110 mV. Ionic current tails increased in amplitude as more channels were activated with a time course that was mirrored by the increase of the fluorescence tail. The exponential fit of data is shown in Fig. 9A as solid lines on the tails of current and fluorescence. Fluorescence tails did not develop as a resolvable peak from the initial fast quenching until after a 20 ms depolarization, but are then plotted compared with the ionic tail current development and fitted to single exponentials (Fig. 9B). The time constants of the fit for fluorescence tails and current activation are shown in Fig. 9C and include the fit of the slow rising phase of fluorescence as well (inset). The data show no significant differences in time constants for the two phases of fluorescence and the ionic tail, suggesting that they track the same underlying event, that is the rate of opening of channels.

**Figure 10:**
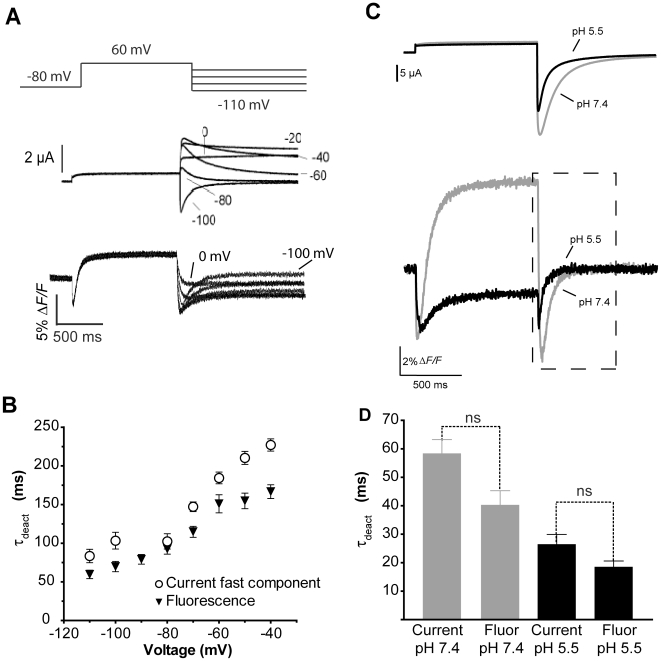
E519C:C445V:C449V tail fluorescence reports on deactivation. (A) Current and corresponding fluorescence reports evoked by a 1 s depolarization to +60 mV from a holding potential of −80 mV, followed by repolarization to potentials ranging from 0 to −110 mV for 2 s. (B) Plot of mean time constants of fluorescence decay fit with a single exponential and the fast time constant of a double exponential fit of current deactivation. (C) Currents (above) and fluorescence (below) evoked by a single pulse to +40 mV from −80 mV at pH 7.4 and 5.5. (D) Comparison of the average time constants obtained using a single exponential fit to the decay of current and fluorescence at pH 7.4 and 5.5. No significant differences were found between time constants of current and fluorescence decay at the two pH's.

In order to further correlate the fluorescence tail signal with channel closing, the rate of channel deactivation over a range of potentials was measured using the protocol shown in Fig. 10A. After a 1 s depolarization to fully activate and inactivate channels, cells were repolarized to potentials ranging from 0 mV to −110 mV to observe deactivation. The rate of decay of the fluorescence signal during the third step matched that of channel deactivation, but exhibited polarity independent of the reversal potential (Fig. 10A). The time constant of decay for both current and fluorescence matched well over the entire range of potentials (Fig. 10B). As a final comparison of the ionic tail current decay and fluorescence tails from E519C:C445V:C449V, a reduction in pH from 7.4 to 5.5 was used as a means of modulating channel deactivation (Fig. 10C). The effects of acidic pH on channel current include a reduction in peak and tail current amplitudes and an increase in the rate of channel deactivation corresponding to a shift in the activation curve [9], [26], [27]. Lowering the pH from 7.4 to 5.5 reduced the amplitude of the upward deflection, consistent with the observed reduction in outward current, and accelerated the time constants of tail current deactivation from 58±4.8 ms at −110 mV to 26.2±3.4 ms (n = 4, Fig. 10D). The rate of decay of the fluorescence tail also became faster at low pH, increasing from 40.3±5.0 ms to 18.3±2.0 ms. The effect of low pH was to accelerate both the rates of ionic current deactivation and the decay of fluorescence tails by 2.2 fold, which further supports the idea that fluorescence quenching during repolarization and its subsequent decay is an excellent measure of pore closure during channel deactivation.

## Discussion

There are four principal findings in this study. The most important is that when endogenous cysteines are removed from the S1–S2 linker, E519C reports a rapid change of S4 voltage sensor environment, seen as a fast initial fluorescence quenching during channel activation gating which has two components. This is the first time that fluorescence has been shown to accurately detect a rapid translocation involving S4 during hERG activation. The second is that the S1–S2 linker cysteines, C445 and C449, both fluoresce when labelled with TMRM, and they modify the overall fluorescence signal from the labelled E519C in the S3–S4 linker. The third finding is that once pore motions occur, they are tracked by E519C and by L520C as the time course and voltage dependence of fluorescence signals upon depolarization and repolarization, during channel activation and deactivation. The fourth finding is that fluorophores placed at E519 and L520 in the S3–S4 linker and at the top of S4 do not track the voltage-dependence or time course of hERG channel inactivation or recovery from inactivation.

### The E519C:C445V:C449V fluorescence tracks fast hERG channel S4 movement

Direct placement of fluorophores at the top end of the S4 voltage sensor and in the S3–S4 linker of voltage-gated channels is usually a reliable way to detect the kinetics of S4 movement during activation. Typically, fluorophores at this site in many channels detect S4 translocation as a rapid quenching of TMRM fluorescence [12], [28], [29], including our own previous data from *Shaker*, Kv1.5, Kv1.4, and Kv1.2 channels [17], [30], which have utilized the A359C *Shaker* site or its homologue. Fluorescence quenching accords with TMRM movement to a more hydrophilic environment, and thus the fast fluorescence quenching we have described from E519C labelling (Figs. 5–7) may be tracking voltage sensor movement outward, as was suggested in other studies using MTSET accessibility [31] or pCMBS [32]. In *Shaker* A359C, and in Kv1.5 channels at A379C, voltage-dependent fluorescence quenching is predominantly fast and monotonic upon depolarization and the *F-V* relationship overlays [17] or approximates [12] the *Q-V* relationship, which is approximately 20 mV hyperpolarized with respect to the *G-V* relationship.

In our study, the initial quenching *F-V_ON_* formed a double Boltzmann relationship, with a *V_½_*_,1_ of −38 mV, and a *V_½_*_,2_ of +44 mV. The component at negative potentials is shifted by −20 mV compared to the *G-V* (Fig. 5), which is comparable with the difference between the *Q-V* and the *G-V* relationship in hERG [4]. As well, the data in Fig. 6 clearly demonstrated a voltage-dependent fluorescence signal from S4 at potentials much more negative than those required to open the pore. Together, these data suggest that the fast initial fluorescence quenching from the labelled E519C:C445V:C449V channel may provide an accurate report of a rapid environmental change at the top end of S4 in hERG channels that signals channel activation. These results are consistent with recent work using the thiol modifying reagent pCMBS which showed that the extent of exposure of S4 in hERG during depolarization is similar to other Kv channels and KvAP [32].

In the only other fluorescence study carried out using E519C, the quenching observed on depolarization was likely affected by the presence of endogenous cysteines since the *V_½_* of the *F-V* relation was found to be +15.6 mV compared with a *V_½_* of the *G-V* relation of −21 mV [11]. Given the very positive voltage-dependence which was inconsistent with activation kinetics, E519C quenching was ascribed to rapid inactivation of hERG channels, but the use of an inactivation-removed mutant (G628C:S631C) did not confirm this hypothesis. Our attempts to correlate the E519C:C445V:C449V fluorescence signal to inactivation were unsuccessful (see Fig. S1). Using either a double- or a triple-pulse protocol, the fluorescence signal was clearly slower than inactivation and did not correlate with the voltage dependence of steady-state inactivation. Indeed, the *V_1/2_* obtained with the double-pulse protocol was −20.3±4.4 mV, which is much more positive than the *V_1/2_* of current obtained using this same protocol in WT hERG channels (about −80 mV) [33]–[36]. The *V_1/2_* obtained using a triple-pulse protocol [37] was −31.8±4.8 mV, again more positive to the one usually obtained with this protocol for WT (about −80 mV) [11], [37]–[40] or for E519C mutant (−90.9 mV) [11]. This is consistent with several studies that show that activation and inactivation are not coupled in hERG [31], [40]–[42]. Our data using the S620T:E519C:C445V:C449V inactivation-removed mutant (Fig. 7) show a rapid initial fluorescence quenching identical with that of E519C:C445V:C449V at activating potentials (compare *F-V* relations in Fig. 7C), and so reinforce our belief that the fast initial quenching of the E519C fluorescence signal upon depolarization represents activation S4 movements, rather than inactivation.

Interestingly, studies with S620T removed the second Boltzmann component of the *F-V_ON_* relation, and the voltage dependence of this environmental change at the top of S4 is similar to that of a fast component of on-gating charge (*Q_On_*) described in earlier studies [4]. This was ascribed to inactivation when first reported, but this hypothesis was later rejected in favour of early transitions in the activation pathway [10]. Interestingly, while the fast *Q_On_* was conserved in an inactivation-removed mutant (S631A) [4], we found that the second component of the double Boltzmann was absent in fluorescence from the E519C:S620T mutant. An explanation for this discrepancy could be the use of different mutants in the two studies. Indeed, S620T mutant abolishes inactivation [25], [43] while S631A mutant induces a depolarizing shift in the steady-state inactivation relation [40]. Thus, further experiments are required to fully understand voltage-sensing of inactivation and the nature of this emission component. Future studies using E519C will enable a greater understanding of conformational changes underlying voltage sensor movements as well as dissection of the gating transitions that contribute to hERG channels' unique drug sensitivity and unusual current behavior.

### Other components of the E519C:C445V:C449V fluorescence and L520C fluorescence signal track channel pore opening and closing

The peak *F-V_OFF_* from the fluorescence tail of E519C has a very similar voltage-dependence and slope factor to the *G-V* relationship (Figs. 5B and 6B), and the envelope of tails test indicated that both the development of the peak of the fluorescence tail and also the fluorescence increase during the maintained depolarization closely matched the time constants of ionic current activation (Fig. 9). As well, the *V_½_* of the *G-V* relation obtained from the ionic tail currents of the inactivation-removed S620T:E519C:C445V:C449V mutant was depolarized to +24.9 mV, and was paralleled by a shift in the fluorescence tail *V_½_* to +18.4 mV (Fig. 7B). Finally, acidic pH, which is known to accelerate hERG current deactivation [9], [27] produced an identical 2.2-fold acceleration of the fluorescence tail decay rate and ionic current tail deactivation (Fig. 10). Taken together these findings strongly support the idea that the tail fluorescence amplitude tracks the number of activated channels, and the time course of fluorescence decay closely reflects channel deactivation.

In the experiments with L520C:C445V:C449V (Fig. 8), the rapid initial quenching seen with E519C was absent, and changes in fluorescence emission occurred with a time course and a voltage dependence identical to ionic current activation, based on the overlay of the *F-V* relationship with the *G-V* (Fig. 8B). L520 is only one position deeper in S4 than E519, but the rotated position in the helix may explain why the fluorophore attached at L520C only senses conformational changes occurring during opening and closing of the channel as a result of its close relationship to the outer pore in this channel. Recent structural work based on Kv1.2 channels places the corresponding residue near the voltage sensor-pore interface within the channel, which may allow it to sense relative movement between the two domains [44]. In agreement with this, recent work has pointed to a critical role of the extracellular S5-P linker in channel activation [35]. Thus, an attractive interpretation of the overlap of the L520C *G-V* and *F-V* would be that when attached to this residue, TMRM at the top of S4 senses a local rate-limiting rearrangement occurring in the extracellular region of the channel pore.

### Possible explanations for slow pore gating in hERG

Based on our results, we propose that following a depolarizing step, the S4 segment moves fast, representing an early step in hERG channel activation. This change allows subsequent rearrangements that result in slow pore opening, which we show here are also tracked by the fluorescence signal at the top of S4 at E519 (Fig. 9) or L520 (Fig. 8). We believe that the two components of the E519C fluorescence signal are related to two separate reorganizations of channel conformation. The fact that they both show substantial voltage-dependence is explained by the fact that they are ‘series-coupled’ events, so that the second movement, which we believe reflects structural reorganization associated with pore opening, has to follow gating charge movement, and gains its voltage dependence from that movement, as in all Kv channels. The slower changes might be due to electrostatic interactions between transmembrane domains, such as S2 and S4 [45], [46] inside the channel structure, since there are three negative charges in S2 of hERG and six overall in the voltage sensor, compared with only two in S2 and four overall in *Shaker* (not counting linker negative charges). It is also possible that the slow gating in hERG might be dependent on turret conformational changes. Indeed, the S5-P linker in hERG is much longer than in other Kv channels, and models suggest that this part of the channel is flexible and far from the pore [47]. Thus, an attractive theory would be that the slow change in fluorescence does not result from S4 movement per se, but rather rearrangements of the adjacent outer pore, which in that case would be responsible for slow activation. S620T is a mutation that removes inactivation, and that is suspected to introduce conformational changes in the outer pore region in hERG. In agreement with this idea, in the inactivation-removed mutant, the fluorescence upward deflection is clearly reduced at all potentials studied (Fig. 7), meaning that, during depolarizations, TMRM attached at E519C was less able to detect environmental changes associated with pore opening in S620T. Either the movements of the turret are reduced in the S620T mutant, or the proximity of the turret to the S3–4 linker is altered, which results in a changed environment around E519C during depolarizations and a reduced detection of outer pore motion.

## Supporting Information

Figure S1 The fluorescence signal from top of S4 does not correlate with inactivation. (A) The same protocol as in Figure 9A was used to measure amplitudes of fluorescence signal upon hyperpolarization (arrow), when channels have recovered from inactivation. These amplitudes were plotted against voltage and normalized. The V1/2 of this fluorescence component was −20.3±4.4 mV (n = 15). (B) Current and corresponding fluorescence reports evoked by a three-pulse protocol. Membrane was depolarized for 1 s to +40 mV from a holding potential of −80 mV, then repolarized to −100 mV for 30 ms, to allow for recovery from inactivation, and stepped again to +40 mV. An overlap of the normalized signals shows that the fluorescence signal (gray) is slower than the current observed from channels that have recovered from inactivation (black). The time constants obtained using single exponential fits are indicated. (C) A three-step protocol was applied to assess the voltage-dependence of fluorescence signal upon the second depolarisation (arrow). Cells were depolarized to +40 mV from −80 mV, hyperpolarized to a range of potentials, and depolarized again to 0 mV. The amplitude of the fluorescence signal upon depolarisation was measured and plotted against voltage (V1/2 = −31.8±4.8 mV (n = 3)).
(0.69 MB TIF)
