# Supplementary figures and images for: Fluorescence-Tracking of Activation Gating in Human ERG Channels Reveals Rapid S4 Movement and Slow Pore Opening
Source: PLoS One. 2010 May 28;5(5):e10876. doi: 10.1371/journal.pone.0010876 (PMC2878317; doi:10.1371/journal.pone.0010876)

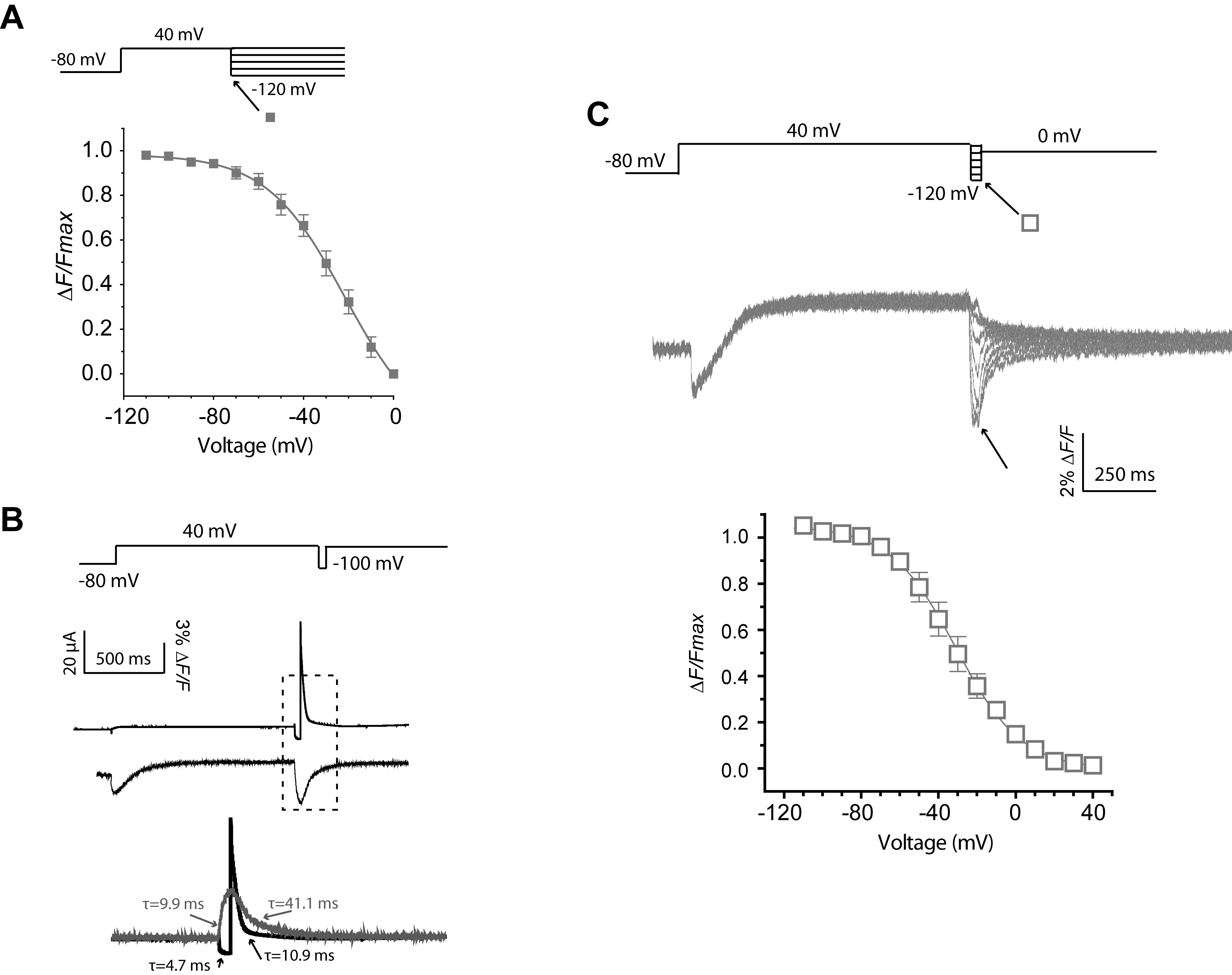

Supplement: Figure S1 — The fluorescence signal from top of S4 does not correlate with inactivation. (A) The same protocol as in Figure 9A was used to measure amplitudes of fluorescence signal upon hyperpolarization (arrow), when channels have recovered from inactivation. These amplitudes were plotted against voltage and normalized. The V1/2 of this fluorescence component was −20.3±4.4 mV (n = 15). (B) Current and corresponding fluorescence reports evoked by a three-pulse protocol. Membrane was depolarized for 1 s to +40 mV from a holding potential of −80 mV, then repolarized to −100 mV for 30 ms, to allow for recovery from inactivation, and stepped again to +40 mV. An overlap of the normalized signals shows that the fluorescence signal (gray) is slower than the current observed from channels that have recovered from inactivation (black). The time constants obtained using single exponential fits are indicated. (C) A three-step protocol was applied to assess the voltage-dependence of fluorescence signal upon the second depolarisation (arrow). Cells were depolarized to +40 mV from −80 mV, hyperpolarized to a range of potentials, and depolarized again to 0 mV. The amplitude of the fluorescence signal upon depolarisation was measured and plotted against voltage (V1/2 = −31.8±4.8 mV (n = 3)). (0.69 MB TIF) [file pone.0010876.s001.tif]
